# Culture and Context in Mental Health Diagnosing: Scrutinizing the DSM-5 Revision

**DOI:** 10.1007/s10912-017-9501-1

**Published:** 2017-12-28

**Authors:** Anna Bredström

**Affiliations:** Institute for Research on Migration, Ethnicity and Society (REMESO/ISV), Linköping University, SE-601 74 Norrköping, Sweden

**Keywords:** DSM-5, Culture, Psychiatry, Depression, International health

## Abstract

This article examines the revision of the *Diagnostic and Statistical Manual of Mental Disorders* (DSM-5) and its claim of incorporating a “greater cultural sensitivity.” The analysis reveals that the manual conveys mixed messages as it explicitly addresses the critique of being ethnocentric and having a static notion of culture yet continues in a similar fashion when culture is applied in diagnostic criteria. The analysis also relates to current trends in psychiatric nosology that emphasize neurobiology and decontextualize distress and points to how the DSM-5 risks serving as an ethnic dividing line in psychiatry by making sociocultural context relevant only for some patients.

## Introduction

In May 2013, the current version of the *Diagnostic and Statistical Manual of Mental Disorders* (DSM-5) was published by the American Psychiatric Association (APA) after more than a decade’s intense discussion both within and outside the professional community engaged in mental health diagnosing. With the aim of improving the previous manual, DSM–IV (1994), an exhaustive revision of concepts and criteria had been carried out since the beginning of the millennia. Among the major issues at stake was what impact current developments in neurobiology would have on the structure and content of the manual. Another issue that hit the news was how DSM-5 got rid of the so called “bereavement exclusion” that existed in DSM-IV and that prevented people in grief after the loss of someone close from being diagnosed with Major Depression Disorder (Zisook et al. 2012). This led critics to raise their voice against what they saw as a decontextualisation of mental distress and an expanding medicalization of normal sadness (Cosgrove and Wheeler 2013; Frances 2013; Wakefield 2013; Whooley 2014).

Ending up somewhat in the shadows of the questions of neurobiology and medicalization, the manual also sought to advance its validity worldwide by being applicable across ethnic and cultural divisions. In their presentation of *Cultural Concepts in DSM-5*, the American Psychiatric Association (2013b) points out that the fifth edition “incorporates a greater cultural sensitivity throughout the manual,” a point that was also emphasised by the Task Force when the new manual was released (Kupfer et al. 2013). In this article, I scrutinize this alleged attempt at making the manual more attuned to cultural differences. The article reveals that, despite its attempt to adopt a more dynamic concept of culture, much of the critique that was raised against the Cultural Formulation of DSM-IV is still relevant for today’s version, i.e. that the manual is ethnocentric and rests upon a narrow understanding of culture (e.g. Lopez and Guarnaccia 2000). In addition to examining the concept of culture as such, I will also point to the ways in which the culture-related diagnostic issues in the manual depart from the manual’s categorical, symptom-based approach which pays minimal attention to context, and, by way of conclusion, I argue that the present manual runs the risk of making context an “ethnic dividing line” between those seen as culturally “other” and those who are not. The article’s contribution to a medical humanities audience is that it explores the intersection of culture and psychiatry (Basset and Baker 2015) and examines an influential medical tool from a sociocultural perspective (Jutel 2009).

## Mode of procedure

The article is based on a critical reading of both the newly released version of the manual, DSM-5 (2013), and its forerunner, DSM-IV (1994), as well as the review reports and statements from the work and study groups assigned to evaluate cross-cultural aspects of DSM-IV and to suggest possible changes for DSM-5 (e.g. Alarcón et al. 2002; 2009; Allik 2006; Ashton and Lee 2005; Becker 2007; Brown and Lewis-Fernández 2011; Escobar and Vega 2006; Hinton and Lewis-Fernández 2011; Kirmayer and Sartorius 2007; Kupfer et al. 2002; Lee and Kleinman 2007; Lewis-Fernández et al. 2010; Regier et al. 2011; Room 2006). During the revision, the cultural expertise reviewed all relevant research published since DSM-IV was released in 1994. They also made secondary analyses of international data and conducted new field trials (Lewis-Fernández et al. 2016).

The DSM-5 revision was in many ways an open and democratic process. Guided by a Task Force appointed by the American Psychiatric Association, it involved numerous experts organised in different subcommittees and work groups, each assigned the task of reviewing and revising criteria for a specific disorder. The review process was also made publicly available through a website, www.DSM-5.org, where drafts of diagnostic criteria and proposed changes were published and open for commentary. Feedback was reviewed by the different work groups, and when possible, incorporated in revised drafts. Finally, criteria for twenty-three disorders were tested through field trials which aimed to evaluate their reliability, feasibility and clinical utility (American Psychiatric Association 2013a). Both field trials and public commentary proved to be important for the final version (American Psychiatric Association 2013a; Whooley 2014; 2016).

With all these experts involved, DSM-5 is in many ways a committee document, which may explain the inconsistencies in understandings and definitions that this article has in its searchlight. However, while the manual is co-authored, it is still *read* as representing one voice and not just any voice but one of the most authorized understandings of mental illness. Throughout the years, DSM has also gained increased importance globally – evident in the on-going process to conform the DSM and the World Health Organisation’s *International Statistical Classification of Diseases and Related Health Problem* (ICD) – which increases its importance even more. Moreover, as has been argued by Bowker and Star (1999), multiple voices and layers of difference are embedded in most classificatory systems, as these often work as “sites of political struggles.” The task for research, thereof, is to map out inherent tensions (Bowker and Star 1999,196), and my ambition in this article is to analyse frictions in the ways culture and context are articulated in key documents of the revision.

Between the two manuals, I have analysed DSM-5 in more depth. Here I have closely studied all chapters and carried out both a quantitative and qualitative content analysis of how culture is addressed in the manual (Bredström 2016). The article focuses on the abovementioned claim of having made DSM-5 more sensitive to cultural issues. The analysis thus juxtaposes the way culture is defined and used in the previous manual (DSM-IV) with the current edition (DSM-5). The article also explores the reviews and statements made by the cultural expertise, focusing on both their critique and suggested revisions, and on their own conceptualizations of culture.

The analysis tackles three cases in point. First, I look at the conceptualization of culture. Here I compare and point to discrepancies between how culture is *defined* and how it is then subsequently *used*. Secondly, I analyse the way psychiatric distress is, or is not, culturally contextualized. Here, I take criteria for Panic Anxiety as an illustrative example of how the manual continues to construct some symptoms of psychiatric distress as *universal*, whereas others are construed of as linked only to *particular* groups of people. In the third part of my analysis, I address the issue of context and its relation to symptoms in the manual more generally by discussing the Cultural Formulation Interview, which is a DSM-5 interview guide designed to be used in clinical practice. I conclude the article by considering how my analytical points relate to current trends in (bio)psyhicatry and mental health diagnosing. Before pursuing my analysis, however, I will first situate DSM in a larger context and present the cultural perspectives of the manuals in more detail.

## DSM and the hegemony of biological psychiatry

At first glance, the fifth edition of the *Diagnostic and Statistical Manual of Mental Disorders* provides a neat impression where disorders are listed chapter by chapter in a menu-like format that contributes to a sense of overview. Looking closer however, one soon gets, as Ian Hacking (2013) expressed it, “lost in the forest.” Lists of symptom criteria are followed by lists of modifying aspects, among others: specifiers to capture additional symptoms not part of current criteria; common comorbidities; differential diagnoses; culture and gender aspects. To keep track of what becomes endless choices of paths, clinicians might turn to the vast number of references that accompany the manual: books presenting clinical cases (Barnhill 2014); trees of diagnostic differences delineating disorders with similar symptoms from each other (First 2013); diagnostic examination tools (Nussbaum 2013); and in-depth knowledge on special disorders or phenomena of interest. Add to this the numerous experts who have been appointed in the revision process, and it is easy to see that the DSM is an industry in and of itself (Cosgrove and Wheeler 2013). Critics have also pointed to ties between some DSM panel members and the pharmaceutical industry that may have compromised the otherwise relatively open and transparent revision process (Cosgrove and Wheeler 2013; Angell 2011).

The manual is meant to serve both as a guide for clinical assessment and a classificatory system of different mental disorders. It makes use of a phenomenological or categorical approach where criteria for a disorder consist of a number of symptoms that need to be met in order to acquire a diagnosis. To be diagnosed with, for instance, Major Depressive Disorder, DSM-5 states that during the same two-week period one should have experienced five of the then listed symptoms such as: “Depressed mood most of the day, nearly every day […];” “Markedly diminished interest or pleasure in all, or almost all, activities most of the day, nearly every day […];” “Insomnia or hypersomnia nearly every day […].” The criteria also state that at least one of the symptoms should be “either (1) depressed mood or (2) loss of interest or pleasure” (American Psychiatric Association 2013a, 160–161). The categorical approach thus implies that symptoms are described and ordered but not linked to any particular etiology.

The categorical approach was introduced with the third edition of the manual, DSM-III, published in 1980. It represented a break with previous diagnostic systems that were based on Freudian psychoanalytic understandings and categorizations (Horwitz and Wakefield 2007). The aim was to avoid hypothetical theories of etiology (Kupfer et al. 2002, xviii), and instead establish a system of classification that could bridge disagreements on possible causes of mental illness. However, even if DSM-III is presented as nonaligned to any particular philosophy, it has yet been looked upon as a benchmark in the history of biological psychiatry. As several critics have argued, a key reason for installing the categorical approach was to reinstate more credibility to psychiatry in response to the anti-psychiatric movements that flourished at the time (Shorter 2013). Instead of relying upon clinical judgement and experience, which were seen as unreliable and unstable, a more systematic approach based upon research data – and not “opinion” – was proposed, following John Feighner and his colleagues (Rose and Abi-Rached 2013, 120). That Feighner et al. had laboratory studies on their wish-list on how to establish valid research data points to the intimate link between DSM-III and what has been conceptualized by Adele Clarke et al. (2010) as the “biomedicalization” of (western) society. Biocapital plays a central role in biomedicalization, as do dominant discourses of consumerism and individualization, and the technoscientific development. In relation to DSM, Jackie Orr (2010) underlines the importance of computerized informatics systems. Using standardized diagnostic criteria enabled comparisons of disorder prevalence and incidence (i.e., epidemiology) on a global scale.

With DSM-5, the advantage of bio-psychiatry is no longer a hidden endeavour. That is to say, while a scientific medical model inspired both DSM-III and DSM-IV, they did not incorporate etiology into the classification scheme. DSM-5 had an explicit aim to break this trend. From the very beginning of the revision process, the DSM-5 Task Force emphasised how the developments in cognitive neuroscience, brain imagining, epidemiology, and genetics bring new light to psychiatry and that such knowledge should impact the revised manual. The goal was to increase validity of psychiatric diagnoses and break with the categorical system of previous editions (Whooley 2014; 2016). As an alternative, the Task Force proposed a dimensional model built upon a “neuroscience-based framework […] in which disorders are grouped by underlying pathophysiological similarities rather than phenomenological observations” (Kupfer and Regier 2011, 2). A dimensional model, they argue, is more in line with the rest of medicine (Kupfer et al. 2013). It also solves the problem of the large number of comorbidities and “not-otherwise-specified” categories that followed upon the categorical model since a dimensional model places mental disorders on a “continuum between normality and pathology, a difference in degree rather than kind” (Whooley 2016, 33).

To follow this through, all work groups assigned to suggest revisions of different disorders by the DSM-5 Task Force were initially instructed to look for biomedical evidence and, if possible, revise the manual in line with such thinking (Kupfer et al. 2002). However, the field trials showed that while the psychometrics of a dimensional model may be valuable for research, it does not carry the same weight in clinical practice. Also, as Owen Whooley has shown by interviewing leading experts of the revision, the process was circumscribed by the way the Task Force gave the different subcommittees to much freedom in designing severity scales (2016, 37). This led to a “hodgepodge” of metrics that became very difficult to synthesize in a coherent classificatory system.

Adjusting the manual to a neuroscientific framework thus turned out to be easier said than done (cf. Pickersgill 2014; Cooper 2015). In 2013, when the manual was published, there was still a lack of strong evidence to justify a complete make-over. Save for some exceptions such as organizing the manual according to developmental and lifespan considerations, and limited changes towards a dimensional model for some disorders, the new manual largely remains symptom-based and descriptive in its approach. Put in the light of biomedicalization theories however, it is clear that DSM-5 expresses the blurry line between normality and pathology that characterizes a biomedicalized society (Clarke et al. 2010). Since the categorical model was introduced with DSM-III, critics have cautioned against the pathologization of normal experience (Horwitz and Wakefield 2007). DSM-5 was no exception. During the revision process, critical commentators (including many prominent psychiatric experts) objected to the expansion of new diagnoses, in particular those that affect already vulnerable groups such as children and elderly (Frances 2013). Arguments was also raised against the medical model that, critics said, decontextualizes human suffering and does not pay enough attention to social aspects, among others, issues of culture, race and ethnicity (e.g. British Psychological Society 2013). It is to these issues I will turn next.

## Cultural differences and the DSM

Alongside searching for scientific evidence of underlying biological mechanisms, attending to cross-cultural matters was also set as a priority for the revision and cross-cultural expertise was appointed to all work groups as well as gathered to form a specific study group (Kupfer et al. 2002). The world-wide use of DSM lay behind the rationale of increasing the cultural sensitivity of the manual, and the tasks for the cultural expertise was to address cultural aspects of each disorder, as well as to draw more comprehensive conclusions across all relevant research areas (Kupfer et al. 2002; Lewis-Fernández et al. 2016).

The need to include cultural perspectives had been acknowledged already in DSM-IV (1994), which pointed to how the manual is used in culturally diverse populations in the United States and internationally. At that time, by the end of the eighties, the field of cultural psychiatry had gained ground in psychiatric circles, which influenced the DSM-IV Task Force (Lewis-Fernández et al. 2016). Cultural psychiatry is a research field that aims to merge “anthropological methods and conceptualizations with traditional psychiatric and psychological approaches” in order to investigate cross-cultural aspects of psychopathology (Lopez and Guarnaccia 2000, 571). Research in this field poses a real challenge to psychiatric diagnosing as it shows that not only do symptoms vary across cultures but that culture also affects how disorders are understood, explained and coped with (Kirmayer and Minas 2000). Cultural aspects have, moreover, shown to be of importance in relation to both help-seeking as well as doctor-patient communication (Kirmayer and Minas 2000), and there are indications that culture takes part in creating “specific sources of stress and distress” (Alarcón et al. 2002, 221) and that there might be yet “un-identified cultural factors” that affect, for instance, comorbidity or severity of illness (Alarcón et al. 2002, 222).

Categorizing the research field of cultural psychiatry, Alarcon et al. (2002, 220) identifies three different “strands” starting with the study of asylums and colonialist psychiatrists that identified and defined different “culture-bound syndromes”, syndromes that only exist within a certain, culturally defined, population. A second strand focuses on culturally diverse populations with a main interest in refugees and migrants’ presumed stress from migration and acculturation processes. The third strand includes a more comprehensive investigation of nosology and diagnostic procedures. This strand acknowledges that also psychiatric knowledge and practice are, in themselves, outcomes of specific socio-political, economic and cultural contexts (see also Kirmayer and Minas 2000).

In both DSM-IV and DSM-5, there are traces of all three strands when looking at how cultural aspects are incorporated. DSM-IV specify that culture is addressed in the manual in three different ways:

1. a discussion in the text of cultural variations in the clinical presentations of those disorders that have been included in the DSM-IV Classification
2. a description of culture-bound syndromes that have not been included in the DSM-IV Classification
3. an outline for cultural formulation designed to assist the clinician in systematically evaluating and reporting the impact of the individual's cultural context.
  (American Psychiatric Association 1994, xxiv)

DSM-5 keeps a similar structure as that of DSM-IV. That is to say, DSM-5 has both a short paragraph on Culture-Related Diagnostic Issues presented adjacent to the diagnostic criteria of most of the disorders; an appendix with a Glossary of Cultural Concepts of Distress; and a specific chapter that presents the Cultural Formulation of the manual in more detail. However, in comparison to DSM-IV, cultural aspects are substantially developed. The notion of culture-bound syndromes is, for instance, problematized in DSM-5. In DSM-IV, the culture-bound syndromes are described as locally expressed illnesses that only appear among certain culturally defined groups and are not necessarily understood as pathological in their own cultural context. The former manual also points out that there is no one-to-one relationship between a culture-bound syndrome and a DSM-disorder. This gives the impression that they are construed as something altogether different, which is further corroborated by the way they are placed in an appendix. However, DSM-IV does indicate that some DSM-disorders – such as Anorexia Nervosa – has “been conceptualised as culture-bound syndromes specific to industrialized countries” (American Psychiatric Association 1994, 844). Yet, none of these are included in the appendix; neither are they portrayed as a culture-bound syndrome in the manual’s main section.

By contrast, the new manual states that the usage of syndrome “ignores the fact that clinically important cultural differences often involve explanations of distress rather than culturally distinctive configurations of symptoms” (American Psychiatric Association 2013a, 758.). Instead, DSM-5 separates “cultural syndromes” from “cultural idioms of distress” that, it is argued, do not necessarily involve symptom expression but yet “provide collective, shared ways of experiencing and talking about personal and social concerns” (758). This said, however, DSM-5’s Glossary of Cultural Concepts of Distress are still to be found in an appendix and thus set apart from the main DSM-disorders; for the unversed it might be hard to discern the difference between them and the previous culture-bound syndromes.

As concerns the second strand – that of migration and refugee matters – this also permeates both manuals and frequently refer to migratory stress and acculturation processes as affecting psychiatric distress. The third, more self-reflective approach that turns attention to psychiatric nosology itself, informs the spirit of the manuals’ *Cultural Formulation*. The Cultural Formulation was launched with DSM-IV and included an assessment of the “Cultural identity of the individual;” “Cultural explanations of the individual’s illness;” “Cultural factors related to psychosocial environment and levels of functioning;” and “Cultural elements of the relationship between the individual and the clinician” (American Psychiatric Association 1994, 843–844). The idea was that the Cultural Formulation would help the clinician to evaluate cultural aspects of the diagnostic procedure, including problematizing the norms and culture of the diagnostic context as such. However, during the revision, the cultural expertise concluded that DSM-IV did not do much to assist clinicians in addressing cultural issues in the doctor-patient meeting. The Cultural Formulation chapter in DSM-5 therefore includes a more in-depth discussion on culture as compared to DSM-IV, and in order to facilitate implementation, the work group developed an interview guide entitled the Cultural Formulation Interview. The guide is included in the manual, and complementary modules are published online and further elaborated on in a Video Illustrated Handbook (Lewis-Fernández et al. 2016). Thus, the revised manual does develop the cultural aspects, yet, as we will see, some of the inherent problems linked to the way that culture is conceptualized nevertheless remain.

## Mixed messages

Let us now turn to the analysis of the revision, beginning with the conceptualization of culture. One of the key points that the cultural expertise put forward to the DSM-5 Task Force was that the new manual would need a more dynamic concept of culture than its predecessor. Several of the reviews referred to how DSM-IV rested upon a static understanding which tended to represent “other cultures” as “distorted reflections of our own cultural preoccupations” (Kirmayer and Minas 2000, 439) or “to ‘exotize’ the cultural approach by ascribing it only to ethnic minorities” (Alarcón et al. 2002, 222), and thus to rest upon an “undeclared ethnocentrism” (Alarcón 2009, 133), referring to how it uses western cultural norms as a tacit measure.

In a white paper on culture and psychiatric diagnoses, Alarcón et al. (2002) refer particularly to López and Guarnaccia’s (2000) critical scrutiny of how culture has been conceptualized within the field of cultural psychiatry generally and in DSM-IV and the *World Mental Health Report* from 1996 specifically. López and Guarnaccia argue that the way culture is being defined in DSM-IV as “values, beliefs, and practices that pertain to a given ethnocultural group” indeed has advantages over earlier definitions of culture within the field of cultural psychiatry, in particular those that located certain expressions of distress within a given ethnocultural group. The “values, beliefs and practices – definition” starts to unpack culture they argue and thus acknowledges heterogeneity. That is to say, it shifts the focus from “belonging to an ethnocultural group” to the value-system of the individual (which might or might not be related to a person’s ethnic identity).

This notwithstanding, López and Guarnaccia underline that the “values, belief and practices-definition” still holds major weaknesses. First of all, it “depicts culture as residing largely within individuals” and thus adheres to an understanding that emphasizes the “psychological nature of culture” (2000, 574) that de-emphasizes the importance of social context. Secondly, Lopez and Guarnaccia argue, the “value, belief and practices-definition” tends to portray culture as a static phenomenon rather than a process. And thirdly, López and Guarnaccia point out that despite opening up for a more heterogeneous understanding of ethnocultural groups that acknowledges intra-group variation, the “value, beliefs and practices definition” does not adequately clarify how individuals negotiate and move between different cultural spheres.

What López and Guarnaccia advocate is a more dynamic understanding of culture that pays attention to both the link between culture and people’s social world and to intra-cultural diversity (class and gender aspects in particular). Other cultural experts have also spoken in favour of a more dynamic concept of culture in DSM but have targeted the issue from another angle. Kirmayer and Sartourius (2007), for instance, take to task the way culture is conflated with ethnicity by questioning the ways in which race or ethnicity are being used as proxy for cultural factors. The usage of “crudely defined” ethnic groups such as African American, Hispanic or Asian American in epidemiological studies, does not, they claim “shed much light on the impact of culture on psychopathological processes” (Kirmayer and Sartourius 2007, 832) as even seemingly well-defined ethnic groups are too heterogeneous. “Current anthropological views,” they contend, demand a focus on how the individual makes use of, and negotiates between, cultural resources, rather than simply assuming a set of values shared by all members of an ethnic or racial collective. Similar to López and Guarnaccia, Kirmayer and Sartourius thus argue that culture needs to be understood as a hybrid system of knowledge that is intertwined with power-relations and reproduced through discourse as well as institutions.

Returning to DSM-5, it is obvious that the revised manual strives towards a more dynamic concept of culture. To a much larger extent than DSM-IV, DSM-5 elaborates how culture is to be understood:

*Culture* refers to systems of knowledge, concepts, rules, and practices that are learned and transmitted across generations. Culture includes language, religion and spirituality, family structures, life-cycle stages, ceremonial rituals, and customs, as well as moral and legal systems. Cultures are open, dynamic systems that undergo continuous change over time; in the contemporary world, most individuals and groups are exposed to multiple cultures, which they use to fashion their own identities and make sense of experience. These features of culture make it crucial not to overgeneralize cultural information or stereotype groups in terms of fixed cultural traits. (American Psychiatric Association 2013a, 749)

Further down is also underlined that “Culture, race, and ethnicity are related to economic inequities, racism, and discrimination that result in health disparities,” which may be interpreted as an understanding of culture, race and ethnicity as socially shaped categories that are intimately related to socio-political matters.

These elaborations on how culture is to be *defined* take place in the specific chapter outlining the manual’s Cultural Formulation, which is located in Section III – “Emerging Measures and Models” – in the manual. Thus one may conclude that as far as the Cultural Formulation goes, the DSM-5 Task Force has attended to the critical remarks summarized above.

At the same time, there are also numerous examples where the critique has not made any visible impact. In fact, looking at the manual as a whole, one is presented with rather mixed messages. The key asymmetry is between Section III and Section II – “Diagnostic Criteria and Codes” where criteria of all DSM-disorders are to be found. Considering Section II, the nuanced and well informed Cultural Formulation found in Section III seemingly loses its weight. Nowhere among the scattered comments on how culture is to be accounted for in relation to specific disorder criteria is it underlined that cultures are to be seen as “open, dynamic, systems that undergo continues change” the way it is expressed in the manual’s own definition quoted above. Neither is it explained how a dynamic understanding of culture as ever changing and heterogeneous would affect the diagnostic procedure itself.

There are, to be fair, some cases that correspond to the spirit of the revision. Let us, for instance, look at how cultural related aspects of Major Depression Episode was formulated in DSM-IV:

… in some cultures, depression may be experienced largely in somatic terms, rather than with sadness or guilt. Complaints of ‘nerves’ and headaches (in Latino and Mediterranean cultures), of weakness, tiredness, or ‘imbalance’ (in Chinese and Asian cultures), of problems of the ‘heart’ (in Middle Eastern cultures), or of being ‘heartbroken’ (among Hopi) may express the depressive experience. […] (American Psychiatric Association 1994, 324)

Compare this paragraph to the corresponding part in DSM-5 that underlines that

[w]hile […] findings suggest substantial cultural differences in the expression of major depressive disorder, they do not permit simple linkages between particular cultures and the likelihood of specific symptoms. Rather, clinicians should be aware that in most countries the majority of cases of depression go unrecognized in primary care settings […] and that in many cultures, somatic symptoms are very likely to constitute the presenting complaint. (American Psychiatric Association 2013a, 166)

Apparently, the authors of this section have explicitly tried to avoid making “other” cultures tantamount to “distorted versions” by emphasising both similarities and problems with linking certain symptoms to particular groups. Also, highlighting the fact of somatization as a general expression of depression could be interpreted as a response to the critique of how western psychiatry tends to incorrectly assign somatization of psychiatric distress as mainly a non-western phenomenon (Kirmayer and Sartorius 2007).

However, looking at Section II as a whole, these examples turn out to be the exceptions to the rule. In general, and in sharp contrast to the dynamic view of the Cultural Formulation, the concept of culture in Section II figures as a static and homogenous group identity that corresponds very much to the “values, beliefs, and practices that pertain to a given ethnocultural group.” criticized by López and Guarnaccia. Under the heading “Culture-Related Diagnostic Issues” of Panic Disorder in DSM-5 one reads for instance that:

[t]he rate of fears about mental and somatic symptoms of anxiety appears to vary across cultures and may influence the rate of panic attacks and panic disorder […]. Also, cultural expectations may influence the classification of panic attacks as expected or unexpected. For example, a Vietnamese individual who has a panic attack after walking out into a windy environment (*trúng gió;* ‘hit by the wind’) may attribute the panic attack to exposure to wind as a result of the cultural syndrome that links these two experiences, resulting in classification of the panic attack as expected. Various other cultural syndromes are associated with panic disorder, including *ataque de nervios* (‘attack of nerves’) among Latin Americans and *khyâl* attacks and ‘soul loss’ among Cambodians … (American Psychiatric Association 2013a, 211)

Here, the “crude categories” (e.g. “Latin Americans,” “Cambodians”), criticized by Kirmayer and Sartourius, that conflate culture with race, ethnicity or nation, or “stereotype groups in terms of fixed cultural traits” (American Psychiatric Association 2013a, 749), are apparently still being used. Moreover, there are no comments on differences relating to class, gender, age, sexuality, migration experience, minority/majority position, transnational and diaspora context and so on.

## Still ethnocentric

The abovementioned critique by the commissioned cultural experts focused not only on that culture needs be conceptualised as dynamic and heterogeneous but also disputed the way DSM-IV took the DSM-diagnoses as self-evident points of departure in a rather ethnocentric manner (e.g. Alarcón et al. 2009). Thus criteria for the DSM-disorders in DSM-IV, the experts argued, were represented as “universal,” unaffected by cultural context, whereas cultural aspects were related only to that which fell outside of the DSM-disorders.

During the revision, there was a time where it looked as if the new manual would take a larger grip on culture and show more reflexivity by culturally contextualize all symptoms and not just some. For instance, the leaflet describing *Cultural Concepts in DSM-5* was published at the DSM-5 website some time before the new manual was out, and it announced that

uncontrollable crying and headaches are symptoms of panic attacks *in some cultures*, while difficulty breathing may be the primary symptom *in other cultures*. (American Psychiatric Association 2013b, my italics)

However, turning to the manual itself, this reflexive outlook did not permeate the final version. Let us take Panic Disorder as an illustrative example of how the manual still can be seen to adopt an ethnocentric approach. The Diagnostic Criteria (A) for Panic Disorder in DSM-5 states that the patient should have

Recurrent unexpected panic attacks. A panic attack is an abrupt surge of intense fear or intense discomfort that reaches a peak within minutes, and during which time four (or more) of the following symptoms occur:

Note: The abrupt surge can occur from a calm state or an anxious state.

- Palpitations, pounding heart, or accelerated heart rate. Sweating.
- Trembling or shaking.
- Sensations of shortness of breath or smothering.
- Feelings of choking.
- Chest pain or discomfort.
- Nausea or abdominal distress.
- Feeling dizzy, unsteady, light-headed, or faint.
- Chills or heat sensations.
- Paresthesias (numbness or tingling sensations).
- Derealization (feelings of unreality) or depersonalization (being detached from oneself).
- Fear of losing control or “going crazy”.
- Fear of dying.

Note: Culture-specific symptoms (e.g., tinnitus, neck soreness, headache, uncontrollable screaming or crying) may be seen. Such symptoms should not count as one of the four required symptoms.

(American Psychiatric Association 2013b, 208)

Accordingly, while shortness of breath passes as one of the (at minimum) four symptoms that would assign a patient the diagnosis Panic Disorder, uncontrollable crying would only count as an additional symptom. In other words – and contrary to what was pledged by the leaflet – shortness of breath is seemingly presented as a *universal* symptom of Panic Disorder, while uncontrollable crying remains a *culturally specific* expression. Thus, the DSM-disorder still subscribes to the non-contextualized point of departure.

This is not to say that the DSM-5 Task Force has ignored the critique of DSM-IV for being ethnocentric. On the contrary, in comparison to DSM-IV, DSM-5 has expanded criteria to include additional symptoms and thus made the disorder more applicable worldwide. For instance, as seen above, criteria for Panic Disorder include a note that states that “The abrupt surge [of a Panic attack] can occur from a calm state or an anxious state.” This note apparently originates from reports showing that the previous requirement that panic attacks should “come out of nowhere” had to be re-thought from a cross-cultural perspective (eg. Lewis-Fernández et al. 2010). Adding to the complexity, the manual also comments upon the imbalance of contextualising only some conditions and not all. It declares that “*all* forms of distress are locally shaped, including the DSM disorders” (American Psychiatric Association 2013a, 758). Yet, the DSM-diagnoses have become global in scope “as a result of their clinical and research utility” (758).

*How* culture informs the DSM-disorders is nevertheless still left untold. Thus, despite the attempts, the core problem remains – that is, the DSM-disorders themselves are not subjected to the cultural critique and cultural aspects are still presented as relevant only for “other” cultures. That this, obviously contradictory, approach also permeated the review process is evident when looking at the papers published by the cross-cultural specialists. In the review of Culture and Anxiety Disorders, the authors point out that one of the weaknesses with their appointed task is that they keep the original DSM-IV disorders as points of departure rather than examining “pathological anxiety more generally” (Lewis-Fernández et al. 2010, 2). This, they argue, might have limited the “identification of alternate constructions of anxiety pathology.” In fact, while the work group on cross-cultural issues kept accentuating the importance of acknowledging the constitutive role of sociocultural processes for mental disorders in order to avoid “essentialism, reductionism, and ethnocentrism that can pre-empt detailed inquiry into their complex etiologic pathways” (Alarcon et al. 2009, 559), hardly any of the review papers looked at the DSM-disorders themselves and analysed them in relation to a specific cultural context. Hence, also the cultural expertise was seemingly swayed by the ethnocentric focus.

## Culture and context

The way cultural aspects in DSM-5 are articulated, as I will argue below, may not only be interpreted as an expression of ethnocentrism and of stereotyping certain groups but also *departs from the very logic of the manual itself* and as such it has a wider bearing on psychiatric diagnostic procedures. Let me explain by returning to the discussion psychiatric nosology raised in the introduction of this article.

As I described above, to a large extent, DSM-5 still relies on a categorical, descriptive approach to diagnoses. Diagnostic criteria thus consist of lists of symptoms characteristic for each disorder without commenting on cause, even if – as was also mentioned earlier – neurobiological theories are highlighted as where future evidence will be found.

The issue of how social and cultural context should be accounted for is a much debated topic in psychiatry. Essentially, it relates to the very definition of a mental disorder. As psychiatry still lacks biomarkers to delineate normality from pathology, contextualising symptom expression becomes crucial in order to avoid false positive diagnoses (Wakefield and First 2012; see also Basset and Baker 2015). Accordingly, phrases such as “more than usual” or “less than normal” permeate disorder criteria, as do comparisons between different environments (e.g. school, home, work etc.). Relying on a similar logic, the manual also distinguishes some conditions that cause distress but states that these should *not* be considered mental disorders precisely due to them having an external cause. These include, among others, “relational disorders,” i.e. abuse or problems related to family upbringing, as well as distress caused by social or economic deprivation. All of them are listed in a specific chapter (in Section II) called “Other Conditions That May Be a Focus of Clinical Attention” and often referred to as the V- or Z-codes.

This provided, DSM-5 was nevertheless criticized for moving towards an increasing decontextualization of mental illness (Frances 2013; Whooley 2014). A key topic that was given much media attention during the revision was how DSM-5 pathologized seemingly normal reactions to outer events. This, critics argue, is what has happened with grief. In DSM-IV, there was the so called “bereavement exclusion,” which omitted someone in grief from being diagnosed with Major Depression Disorder. Hence grief was seen as a normal reaction and not an illness. During the review process, critics questioned both the short span that the bereavement exclusion allowed for grief and the narrow understanding of loss that it contained and argued in favour of paying much more attention to context in order not to pathologize normal sadness (Wakefield 2013). The DSM-5 Task Force, however, took another turn and instead eliminated the exclusion all together (Zisook et al. 2012; Regier et al. 2013). To be fair, the Task Force agreed that it was too limited and that other circumstances than grief could cause similar reactions. Their main argument, however, was not that there was too little context but rather the opposite - that context was not relevant for the diagnosis as such (Barnhill 2014). To the contrary, the Task Force and DSM-5 Mood Disorders Work Group argued that the bereavement exclusion could hinder people who develop major depression while grieving from receiving appropriate care (Zisook et al. 2012). Nevertheless, DSM-5 do still comment upon affective differences between grief and major depression and encourages clinicians to pay attention to experiences of loss when judging the symptoms of the patient. Yet, on a broader, political level, abandoning the bereavement exclusion is a strong signal that – together with the increased emphasis on genetics and neuroscience – points to a general disinterest in the sociocultural context where symptoms of distress are experienced and lived (Whooley 2014).

For our purpose here, the interesting issue is that when culture-related aspects are brought up, this narrow focus on “symptoms only” tends to fade into the background, and context becomes much more present. Indeed, acknowledging that culture affects how conditions are experienced, interpreted and explained serves as the very rationale behind incorporating a stronger cultural perspective. In contrast to the way the experts reasoned around grief, the cultural experts tended to emphasise the importance of contextualizing distress:

[T]he type of worry experienced by an undocumented individual in a US setting, after a raid by immigration services, may be deemed ‘excessive’ by a clinician with insufficient knowledge of the contextual factors underlying this worry. This person may ostensibly fulfil Generalized Anxiety Disorder (GAD) criteria and yet not be suffering from the same psychiatric disorder (or a disorder at all), as someone without these contextual factors. (Lewis-Fernández et al. 2010, 214)

An illustrative example of how cultural aspects focus context is the Cultural Formulation Interview (CFI). As mentioned earlier, to meet the critique about DSM-IV’s Cultural Formulation being difficult to implement in the clinical setting (Alarcon et al. 2009), the CFI was developed. It is an interview guide designed to assess cultural aspects of mental illness. It is located in the chapter on culture in Section III of the manual, and supplementary modules are published online and further developed in the *DSM-5 Handbook on the Cultural Formulation Interview* (Lewis-Fernández et al. 2016). The CFI urges the clinician to become an ethnographer and use the interview guide to capture the patient’s own perspectives. Several of the questions focus on how the patient and his/her community understand the illness:

People often understand their problems in their own way, which may be similar to or different from how doctors describe the problem. How would *you* describe your problem?

Sometimes people have different ways of describing their problem to their family, friends, or others in their community. How would you describe your problem to them?

Some people may explain their problem as the result of bad things that happen in their life, problems with others, a physical illness, a spiritual reason, or many other causes.

Why do you think this is happening to you? What do you think are the causes of your [PROBLEM]?

What do others in your family, your friends, or others in your community think is causing your [PROBLEM]? (Cultural Formulation Interview, American Psyciatric Association 2013a, 752)

These kinds of questions should be compared with diagnostic criteria of the main DSM disorders such as Major Depressive Disorder and Panic Disorder mentioned earlier, which focus on symptoms only – i.e. the “Palpitations, pounding heart, or accelerated heart rate” and “Fear of losing control or ‘going crazy’” of Panic Attacks, or “Markedly diminished interest or pleasure in all, or almost all, activities most of the day, nearly every day” in Major Depression (American Psychiatric Association 2013a, 208; 160–161). That is to say, whereas diagnostic criteria generally focus on symptoms and symptom severity in a decontextualized manner, the CFI is all about contextualising symptoms.

Accordingly, one may conclude that cultural aspects translate into contextualising symptoms of mental distress, but the reverse is also true – that is, to contextualise symptoms becomes equal with cultural difference. Of course, the CFI does not explicitly say that it cannot be applied to all patients, that it could very well be used as a means to investigate the contextual aspect of any patient regardless of ethnic or racial background, which is also what is proposed by some cultural experts (Lewis-Fernández et al. 2016). Yet, the way culture only relates to “otherness” throughout the manual does not suggest that will be the case. And, reading the manual, the cultural “others” of DSM-5 are Latin Americans, Vietnamese and Middle Easterners, not people from a predominantly white western culture.

## Conclusion

In this article, I have examined the claim of DSM-5 incorporating a “greater cultural sensitivity” (American Psychiatric Association 2013b). In sum, my argument is that this is true to the extent that DSM-5 explicitly addresses some of the drawbacks inherent in DSM-IV’s approach to culture. Yet, other shortcomings remain, and in the end DSM-5’s approach appears rather skewed: What we have is a manual that acknowledges that culture affects all disorders yet only presents some symptoms as culturally contextualized. And, similarly, we have a manual that rests upon a dynamic definition of culture, but which in its application nevertheless falls back on a very static notion of culture. Finally, we have a manual that cautions against stereotyping and the employment of simplistic connections between a group identity and a certain condition but then repeatedly uses broad categories – be they Latin Americans, “societies with collective norms” or “Vietnamese” – in a fashion very similar to the one it explicitly said it would jettison.

To a certain extent, such “mixed messages” can be explained with reference to the fact that the DSM-5 is a committee document, co-authored by numerous experts in different fields. Still, this does not make a sociocultural critique less relevant. To the contrary, critics have called for an increasing scholarly engagement in the “Sociology of diagnosis” (Jutel 2009; see also Pickersgill 2014), as diagnoses are powerful apparatuses of medicine today. Diagnoses guide treatment as well as funding and have vast impact upon how illness is experienced, understood and lived. Psychiatric diagnoses are perhaps even more salient than other medical diagnoses, as they centre on a person’s inner life, feelings and behaviours. Critics have also warned against diagnostic inflations in disorders such as ADHD, following the increasing authority of DSM (Frances 2013).

The mixed messages above may also be explained with reference to the categorical system of the manual. A dynamic notion of culture corresponds to the ideals of patient-centred consultations that dominate (western) health policy but is very difficult to adapt to the checklist mode of categorising psychiatric distress. Indeed, the DSM-disorders may themselves be seen as “crude categories” that homogenise and simplify. The suggested move towards a dimensional model does not necessarily solve this problem as it seems likely that it will focus on measuring severity and recognising crosscutting symptoms, rather than locating suffering in its cultural context (see American Psychiatric Association 2013a, 733–748; Whooley 2014).

In this article, the final argument demonstrates that the cultural approach – as it is articulated in the manual – has effects beyond being ethnocentric and simplistic. With DSM’s strong emphasis on decontextualizing symptoms and its aspirations of establishing neurobiological classification schemes, the cultural approach stands out as it keeps putting social, cultural, economic and political context to the fore. Rather than DSM-5 having become more attuned to cultural context, this article has shown that context within DSM-5 becomes an ethnic dividing line between those who are seen as culturally “other” and those who are not. Thus, instead of attending to how symptoms are lived and interpreted in a particular cultural context, both culture and context interchangeably become reduced to a marker of identity. Following DSM-5, assessing cultural aspects in the clinical setting may therefore be an arbitrary affair.
